# Ciprofol-etomidate versus propofol-etomidate for gastrointestinal endoscopy: a randomized, double-blind controlled clinical trial

**DOI:** 10.3389/fmed.2026.1714132

**Published:** 2026-04-24

**Authors:** Zhen Gu, Xiaoli Huang, Aolin Ren, Chi Xu, Huiwen Zheng, Yan Zhi

**Affiliations:** Department of Anesthesiology, Wuxi No.2 People’s Hospital (Affiliated Central Hospital of Jiangnan University), Wuxi, China

**Keywords:** ciprofol-etomidate, frailty, hemodynamic stability, propofol-etomidate, sedative safety

## Abstract

**Objective:**

Explore the clinical efficacy and safety of ciprofol-etomidate (C-E) versus propofol-etomidate (P-E) for gastrointestinal endoscopy sedation, and generate hypotheses for subsequent prospective pre-registered trials.

**Methods:**

A prospective, double-blind, randomized, positive-controlled exploratory trial enrolled 240 adults (120 per group). Exploratory outcomes included hemodynamics, adverse events, and Chalder Fatigue Questionnaire (CFQ) scores. Intention-to-treat (ITT) analysis was primary, with per-protocol analysis (PPA, *n* = 223) as sensitivity validation.

**Results:**

C-E group showed significantly lower CFQ Bimodal scores ≥4, postoperative dizziness incidence, and blood pressure fluctuations (>20% baseline; all *p* < 0.05). One P-E patient needed respiratory support for SpO₂ = 85%. Other outcomes (e.g., induction time, total dose) were comparable. ITT and PPA results were consistent.

**Conclusion:**

This retrospective registered exploratory study tentatively suggests C-E may offer potential advantages in hemodynamic stability, respiratory safety, and reduced post-procedural fatigue/dizziness for low-risk (ASA I ~ II) patients. Findings are hypothesis-generating and require validation via prospective pre-registered trials.

**Systematic review registration:**

https://www.chictr.org.cn/indexEN.html, identifier ChiCTR2500107221.

## Introduction

Currently, anesthetic agents used for sedative endoscopic procedures are primarily classified into sedatives (e.g., propofol, remimazolam, etomidate) and analgesics (e.g., the opioid remifentanil) (1). Among these, propofol remains the gold standard for sedation due to its short duration of action and rapid onset and recovery; however, it is still associated with adverse effects such as respiratory depression and significant hemodynamic fluctuation (2). Ciprofol, a novel short-acting *γ*-aminobutyric acid (GABA) receptor agonist and structural analog of propofol, has emerged as a promising alternative. Compared with propofol, it exhibits significantly reduced respiratory depression, minimal injection pain, and improved hemodynamic stability (3, 4). These advantages have led to its increasing application in non-operating room anesthesia, particularly in sedative endoscopic procedures and outpatient procedures such as induced abortion, highlighting its potential as a safer option for procedural sedation. Nevertheless, the recovery time of ciprofol when used for sedative procedures is longer than that of propofol.

The combination of propofol and etomidate is also widely recognized in clinical practice. Among various regimens, the 1:1 volume ratio combination of propofol and etomidate (e.g., 1 mg/kg propofol + 0.2 mg/kg etomidate) has been extensively adopted clinically due to its proven ability to reduce hemodynamic fluctuations and mitigate propofol-induced injection pain, however, it is associated with a higher incidence of adverse events including muscle spasms, involuntary body movements, apnea, and swallowing reflex (5–7). Currently, there are few studies on the ciprofol-etomidate combination for procedural sedation, and existing literature lacks direct comparisons of key outcomes between these two regimens—such as sedation quality (e.g., Observer’s Assessment of Alertness/Sedation [OAA/S] scores), procedure-related complications (e.g., hypotension, bradycardia, hypoxemia), and patient-reported outcomes (e.g., satisfaction, recovery quality).

Against this backdrop, this prospective, double-blind, randomized controlled exploratory trial aims to explore the clinical efficacy and safety of ciprofol-etomidate versus propofol-etomidate for sedation during gastrointestinal endoscopy, and to generate hypotheses for subsequent prospective, pre-registered trials. We tentatively propose that the ciprofol-etomidate combination may have potential advantages in reducing hemodynamic fluctuations and postoperative fatigue due to the superior pharmacological properties of ciprofol, providing a basis for further confirmatory research.

## Method

### Ethics

This study was conducted in accordance with the Declaration of Helsinki and approved by the Ethics Committee of Central Hospital Affiliated to Jiangnan University - Wuxi Second People’s Hospital (Approval (2023) Ethical Review No. Y-115). This study was initiated in 2023 and was retrospectively registered with the Chinese Clinical Trial Registry (registration number ChiCTR2500107221; https://www.chictr.org.cn/indexEN.html/) on August 6, 2025, upon recognizing the importance of prospective trial registration. Written informed consent was obtained from all participants. All data were anonymized before analysis and stored in a password-protected database accessible only to the research team. The Education Department of our hospital has designated personnel to supervise the trial process and data confidentiality of projects that have passed ethical review.

### Trial design

This trial adopted a prospective, double-blind, randomized, positive-controlled exploratory design, aiming to explore potential differences in hemodynamic stability and other safety/efficacy indicators between the ciprofol-etomidate (C-E) group and the propofol-etomidate (P-E) group, rather than conducting a confirmatory superiority test. The pilot study showed that the mean arterial pressure (MAP) of patients in the propofol-etomidate combination group (control group) decreased by an average of approximately 25% compared with the baseline during endoscopy. We set the clinically relevant difference for exploratory purposes as “a difference of ≥20 percentage points in the magnitude of MAP reduction between the experimental group and the control group,” which means we tentatively expected the magnitude of MAP reduction in the ciprofol-etomidate combination group (experimental group) to be reduced by 20 percentage points or more compared with the control group, ultimately controlling the magnitude of MAP reduction in the experimental group at 5% or less. To ensure sufficient statistical power for exploratory analysis, a sample size of 108 patients per group was calculated (80% statistical power, alpha error rate of 5%), and considering a 10% dropout rate, the final sample size was set to 120 patients per group.

This study enrolled 240 adult Chinese patients at the Affiliated Central Hospital of Jiangnan University (Wuxi Second People’s Hospital) between October and November 2023. These patients were scheduled to undergo sedated endoscopic examinations and/or sedated endoscopic treatments after preoperative sedation assessment. The cohort included patients undergoing various sedative endoscopic procedures, including diagnostic gastroscopy/colonoscopy, gastroscopic/colonic endoscopic mucosal resection (EMR), and endoscopic submucosal dissection (ESD). Participants were randomly allocated to either the Ciprofol-Etomidate group (C-E group, *n* = 120) or the Propofol-Etomidate group (P-E group, *n* = 120). Patients included in the study were randomly assigned to each endoscopic operating room via computer-generated queuing numbers. All study drugs (ciprofol, propofol, and etomidate) were milky-white solutions indistinguishable in appearance. An independent researcher not involved in clinical management or outcome assessment prepared the medications in a separate room according to the randomization sequence. Each drug was drawn into identical 20 mL syringes labeled only with a numeric code (ciprofol: “No.1,” propofol: “No.2,” etomidate: “No.3”). This coding system and the key were kept strictly concealed from all clinical staff involved in anesthesia and nursing care. The administering anesthesiologist knew only which coded syringe to use per the study protocol but remained blinded to the actual drug identity. Intraoperative and immediate postoperative data were recorded on case report forms by a ward nurse who was also unaware of the code meanings, under the verbal guidance of the anesthesiologist (e.g., “administer 5 mL of drug No.1 now”). Post-procedure follow-up assessments were conducted by trained post-anesthesia care unit (PACU) nurses.

Seven patients did not receive the assigned intervention. The primary reasons were contraindications identified during the pre-anesthesia reassessment upon entering the procedure room—specifically, new-onset arrhythmias or severe hemodynamic instability that precluded safe anesthesia (*n* = 5). Additionally, two patients withdrew consent due to anxiety after the reassessment and declined intervention. Additionally, 10 patients were lost to follow-up during postoperative recovery room monitoring (as they refused to complete the postoperative questionnaire). Consequently, 223 patients completed the study protocol, with 113 in the C-E group and 110 in the P-E group.

Inclusion criteria

1. Eligibility for sedation confirmed through preliminary preoperative sedation evaluation
2. Age of 18–80 years
3. ASA (American Society of Anaesthesiologists) grade I–II
4. Capacity to engage in logical dialogue and address study-related questions pertinently

Exclusion criteria

1. Other contraindications for intravenous anesthesia include conditions such as recent upper respiratory infection or unresolved cough.
2. Documented psychiatric disorders via medical history review

### Study intervention

According to the drug instructions, 1 mL of ciprofol is nearly equivalent to 1 mL of propofol in sedative anesthesia (3, 8). Therefore, the calculation formula for the bolus dose (ml) in both patient groups is weight (kg)*(2 ~ 2.5)/10 mL (derived from the propofol-only dosing regimen). For the C-E group, the initial bolus dose ratio of ciprofol (ml) to etomidate (ml) was 2:1. For the P-E group, the initial bolus dose ratio of propofol (ml) to etomidate (ml) was 2:1. Intraoperative supplemental doses were set at 3 mL (ciprofol/propofol:etomidate = 2:1). In the C-E group, the administration sequence involves first administering ciprofol followed by etomidate, and similarly, in the P-E group, propofol is administered first followed by etomidate. Digestive endoscopy procedures should only be performed after the patient becomes unresponsive to verbal commands and loses the eyelash reflex.

Intervention measures during sedation procedures:

Respiratory depression:

All patients received nasal cannula oxygen inhalation preoperatively (flow rate: 3 L/min). In the event of decreased SpO₂, measures such as airway opening and increasing oxygen flow rate were first implemented in accordance with the protocol. If no improvement is observed, interventions such as respiratory stimulants (e.g., doxapram) and manual ventilation are administered.

Blood pressure fluctuation (>30% Baseline):

Vasoactive agents (e.g., phenylephrine for hypotension, urapidil for hypertension) were administered.

Heart rate (HR) abnormalities:

Bradycardia (HR < 50 bpm): The operator was alerted to pause the procedure, and atropine was administered if unresolved.

Sinus Tachycardia (HR > 100 bpm): Anesthetic depth was increased or beta-blockers (e.g., esmolol) were administered.

**Figure 1:**
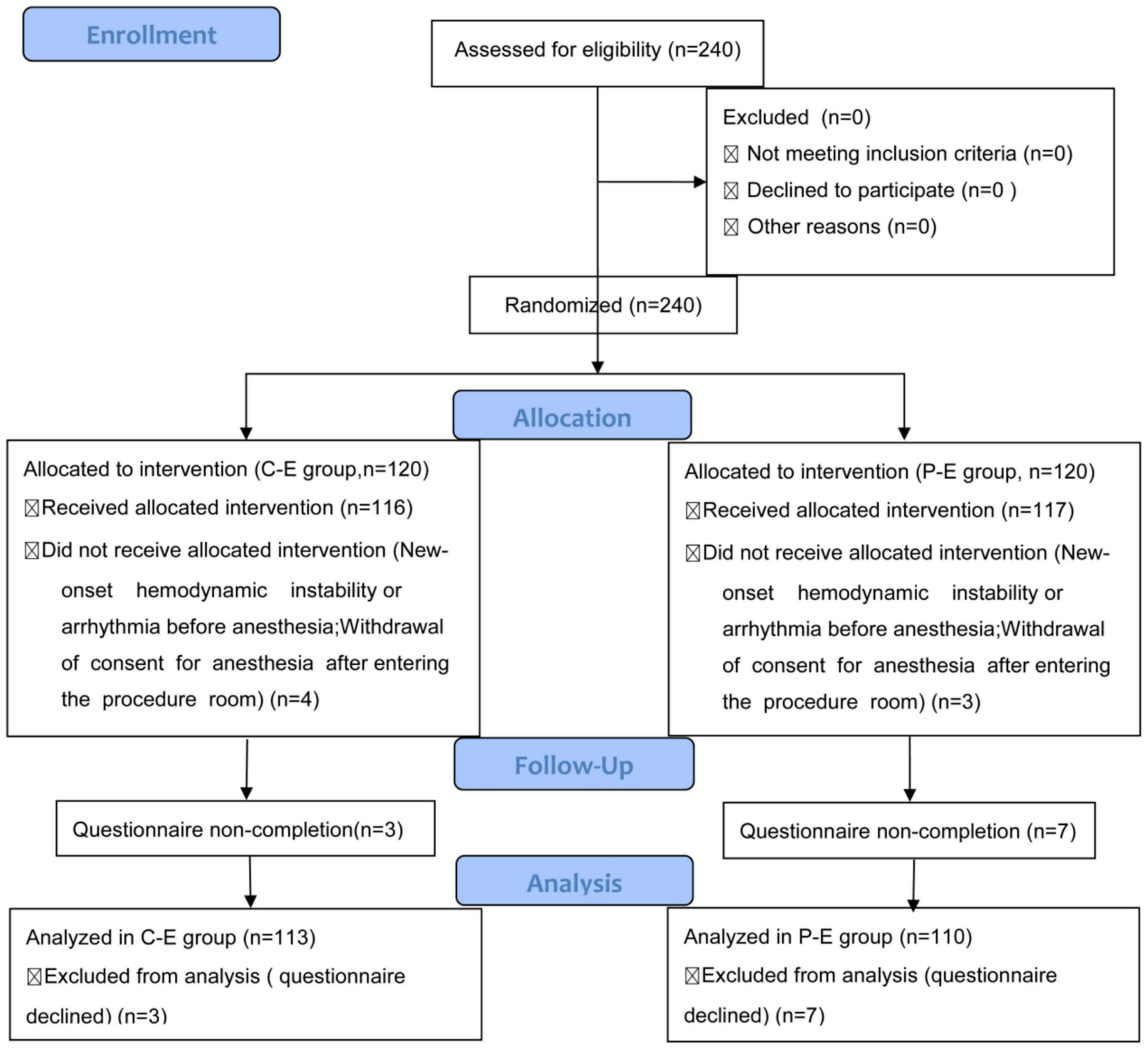
Patient enrollment and follow-up flow diagram. A total of 240 patients were randomized (120 to the C-E group and 120 to the P-E group). After allocation, four patients in the C-E group and three patients in the P-E group did not receive the assigned intervention due to new-onset hemodynamic instability/arrhythmia before anesthesia or withdrawal of anesthesia consent after entering the procedure room. Ultimately, 113 patients in the C-E group and 110 patients in the P-E group completed the study and were included in the analysis; 3 and 7 patients from the respective groups were excluded from analysis due to refusal to complete the questionnaire. C-E, Ciprofol-Etomidate; P-E, Propofol-Etomidate.

The flow chart of this trial is represented in Figure 1.

### Exploratory outcome measures

Routine data collection included general information such as gender, age, height, weight, medical history, and type of procedure. Upon entering the procedure room, three consecutive non-invasive blood pressure (NIBP) measurements were taken, with the lowest reading recorded as the baseline blood pressure. The lowest heart rate observed within 15 min of entering the room was recorded as the baseline heart rate. Patients were continuously monitored for electrocardiogram (ECG), heart rate (HR), non-invasive blood pressure (NIBP), and pulse oxygen saturation (SpO_2_) throughout the procedure. For hemodynamic monitoring, four key phases were defined: T0 (baseline before sedation), T1 (during endoscope insertion), T2 (3 min post-initiation), and T3 (1-min post-procedure). Adverse events in procedural sedation such as hypotension, respiratory depression, use of assisted ventilation equipment, respiratory stimulants, and vasoactive agents were documented. Adverse reactions during and after the procedure—including coughing during gastroscopy, myoclonus, postoperative dizziness, nausea/vomiting, and restlessness—were also recorded. When the patient’s Observer’s Assessment of Alertness/Sedation (OAA/S) score reached ≥4, the Chalder Fatigue Questionnaire (CFQ) was administered (frailty was defined as a “B” score ≥4 or an “L” score ≥18) (9, 10). Patient satisfaction was assessed concurrently.

Exploratory core outcomes:

1. Success rate of sedated endoscopic procedures
2. Hemodynamic parameters (values at each time point and perioperative hemodynamic nadir)
3. CFQ Bimodal and Likert scores when OAA/S score ≥4

Exploratory secondary outcomes:

1. Induction duration and total drug administration duration
2. Presence of severe coughing during gastroscope insertion
3. Occurrence of intense body movements requiring repeated supplemental doses for suppression
4. Intraoperative use of respiratory stimulants
5. Postoperative dizziness or nausea when OAA/S score ≥4
6. Number of supplemental drug doses administered

### Data analysis

All data generated during the study period were anonymized for management and monitoring purposes. Since there is no funding source for this trial, no Data Monitoring Committee (DMC) was established. The principal investigator (PI) was responsible for data management and verification of data accuracy. A total of 233 patients received the trial intervention, and 10 patients refused to complete the questionnaire in the Post-Anesthesia Care Unit (PACU).

Statistical analysis was performed using intention-to-treat (ITT) analysis as the primary approach, which included all 240 randomized patients, to minimize selection bias. Per-protocol analysis (PPA) was conducted as a sensitivity analysis, including 223 patients who completed the entire study protocol, to verify the robustness of the results. It is emphasized that PPA is not considered “better reflecting the effect” than ITT; instead, the consistency between ITT and PPA results is used to support the reliability of the exploratory findings.

Statistical analyses were performed using R software (version 4.2.1). The Shapiro–Wilk test and Levene’s test were applied to assess data normality and homogeneity of variance, respectively. Normally distributed continuous variables were expressed as mean ± standard deviation (SD) and compared using Student’s t-test. Non-normally distributed continuous data were summarized as median (interquartile range, IQR) and analyzed with the Mann–Whitney U test. This study employed a linear mixed model for analysis, with Bonferroni correction applied to control for potential multiple comparison biases. The fixed-effect structure comprised group, time, and their interaction, while the random-effect structure was specified as a patient-specific intercept, aiming to assess changes in hemodynamic parameters over time across groups. Categorical variables were presented as frequencies or percentages and compared using chi-square tests or Fisher’s exact test, as appropriate. A two-sided *p* < 0.05 was considered statistically significant for all analyses.

## Results

### Study population and flow

Two hundred twenty-three patients (C-E group: 113; P-E group: 110) completed the trial (Figure 1). No statistically significant differences were observed between the two groups in any of the metrics, including the type of endoscopic procedure, gender, age, height, weight, history of sleep disorders, long-term use of sedatives, long-term alcohol history, hypertension, diabetes mellitus, long-term smoking history, history of ischemic stroke, duration of the endoscopic procedure, and pre-procedural vital signs (SBP, MAP, heart rate, SpO_2_) (Table 1).

**Table 1:** Basic demographic data and clinical characteristics of the study groups.

Basic demographic data and clinical characteristics	C-E group (*n* = 113)	P-E group (*n* = 110)	*p* value
Type of procedure [Gastroscopy/ Colonoscopy/ Gastrointestinal endoscopy/Colonic EMR/Colonic ESD/Gastroscopy combined with colonic EMR/Gastric EMR/Gastric EMR combined with Colonic EMR/Gastric Endoscopic Ultrasound (EUS)]	2/15/89/3/1/2/0/0/1	1/9/90/4/0/2/2/2/0	0.509
Gender (Male/Female)	51/62	44/66	0.522
Age (y)	58.0 [47.0;64.0]	57.0 [49.0;65.8]	0.691
Height (cm)	163 [158;170]	163 [158;168]	0.891
Weight (kg)	60.0 [55.0;70.0]	60.0 [54.0;70.0]	0.976
Sleep disorders (Yes/No)	13/100	16/94	0.634
Sedatives (Yes/No)	11/102	12/98	0.946
Long-term alcohol history (Yes/No)	6/107	8/102	0.743
Hypertension (Yes/No)	24/89	31/97	0.295
Diabetes Mellitus (DM) (Yes/No)	9/104	6/104	0.631
Long-term smoking history (Yes/No)	13/100	12/98	1.000
History of ischemic stroke (Yes/No)	3/110	2/108	1.000
Endoscopic Procedure Duration (min)	18.0 [15.2;21.5]	17.5 [13.8;20.9]	0.256
SBP (mmHg)	144 ± 16.6	148 ± 18.5	0.189
MAP (mmHg)	102 ± 11.5	103 ± 10.1	0.498
Heart rate (bpm)	81.0 [73.0;88.0]	78.0 [73.2;86.0]	0.586
SpO_2_ (%)	98.0 [97.0;98.0]	98.0 [97.0;98.0]	0.637

This table presents the baseline characteristics of all included patients (*n* = 223). No statistically significant differences were observed between the two groups in any of the metrics, including the type of endoscopic procedure, gender, age, height, weight, history of sleep disorders, long-term use of sedatives, long-term alcohol history, hypertension, diabetes mellitus, long-term smoking history, history of ischemic stroke, duration of the endoscopic procedure, and pre-procedural vital signs (SBP, MAP, heart rate, SpO_2_) (all *p* > 0.05). These results indicate that the baseline characteristics of the two patient groups were comparable.

EMR, endoscopic mucosal resection; ESD, endoscopic submucosal dissection; EUS, endoscopic ultrasound; SBP, systolic blood pressure; MAP, mean arterial pressure.

Categorical data are presented as counts. Normally distributed continuous data are presented as mean ± standard deviation (e.g., SBP, MAP), while non-normally distributed continuous data are presented as median [interquartile range]. *p* values were calculated using the Chi-square test (for categorical variables), Student’s *t*-test (for normally distributed continuous variables), or the Mann–Whitney U test (for non-normally distributed continuous variables).

### Pharmacodynamic profiles and drug consumption in the C-E and P-E groups

Sedation was successfully induced and maintained in all patients across both groups. The volume of the initial bolus dose was comparable between the C-E group (15.0 [12.9–16.2] ml) and the P-E group (15.0 [13.1–17.2] ml; *p* > 0.05). Furthermore, no significant differences were found in the time to induction (C-E: 51.0 [45.0–59.0] s vs. P-E: 56.5 [44.0–68.0] s; *p* = 0.121) or the time to loss of eyelash reflex (C-E: 59.0 [52.0–70.0] s vs. P-E: 65.0 [52.0–75.0] s; *p* = 0.429). Additional procedural metrics, including the number of supplemental doses, total drug consumption, and the interval from the last drug administration to the end of the procedure, were also similar between groups (Table 2).

**Table 2:** Pharmacodynamic profiles and drug consumption in the C-E and P-E groups.

Pharmacodynamic profiles and drug consumption	C-E group	P-E group	*p* value
Induction time (s)	51.0 [45.0;59.0]	56.5 [44.0;68.0]	0.121
Time to loss of awareness (s)	59.0 [52.0;70.0]	65.0 [52.0;75.0]	0.429
First blous (ml)	15.0 [12.9;16.2]	15.0 [13.1;17.2]	0.564
Cumulative dose (ml)	21.0 [18.0;24.3]	21.1 [18.3;24.3]	0.361
Number of supplemental doses	2.00 [1.00;3.00]	2.00 [1.00;3.00]	0.421
Interval from the last drug bolus to end of endoscopic procedure (min)	6.95 [5.17;9.40]	7.08 [5.52;8.89]	0.893

This table compares the sedative induction efficiency and drug usage between the two patient groups. The data show no statistically significant differences were observed between the C-E group and the P-E group in all evaluated metrics, including induction time, time to loss of awareness, initial bolus dose, cumulative dose, number of supplemental doses, and the interval from the last drug bolus to the end of the endoscopic procedure (all *p* > 0.05).

Data are presented as median [interquartile range]. Between-group comparisons were performed using the Mann–Whitney U test (appropriate for non-normally distributed data). *p* values are from superiority tests; non-inferiority margins were not pre-specified. *p* < 0.05 was considered statistically significant.

### Hemodynamic profiles

1. Systolic blood pressure (SBP).

**Figure 2:**
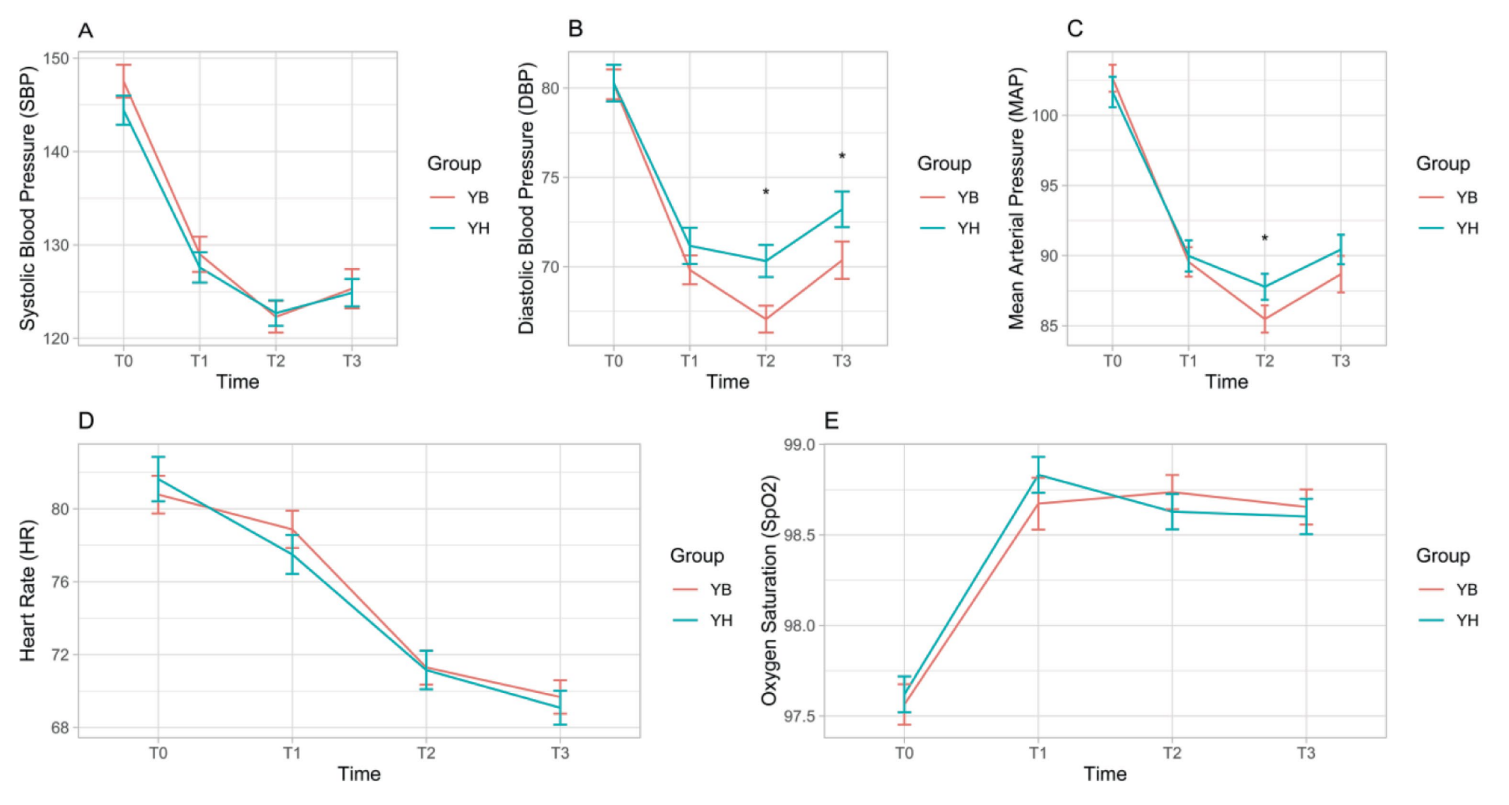
Comparison of vital signs between two groups. Trajectories of mean ± standard error (SE) vital signs (SBP, DBP, MAP, HR, SpO_2_) for the intervention and control groups over follow-up (T0–T3). Changes from baseline (T0) and between-group differences at each subsequent time point (T1–T3) were assessed using a linear mixed-effects model; *p* values are from superiority tests; non-inferiority margins were not pre-specified. *p < 0.05. SBP, systolic blood pressure; DBP, diastolic blood pressure; MAP, mean arterial pressure; HR, heart rate; SpO_2_, peripheral oxygen saturation; SE, standard error. **(A)** Comparison of SBP between two groups. **(B)** Comparison of DBP between two groups. **(C)** Comparison of MAP between two groups. **(D)** Comparison of HR between two groups. **(E)** Comparison of SpO_2_ between two groups.

The estimated marginal means of SBP were 130 mmHg (95% CI: 127–133) in the C-E group and 131 mmHg (95% CI: 128–134) in the P-E group. No significant main effect of group (*p* = 0.572) or group × time interaction (*p* = 0.389) was observed (Figure 2A; Table 3).

2. Diastolic blood pressure (DBP).

**Table 3:** Estimated marginal means and differences between the two groups.

Hemodynamic parameters	Group		Difference	*p* value
	C-E	P-E		
SBP (mmHg)	130 [127133]	131 [128134]	−1.14 [−5.08 2.81]	0.572
DBP (mmHg)	73.7 [72.3 75.2]	71.9 [70.4 73.3]	1.88 [−0.18 3.88]	0.066
MAP (mmHg)	92.5 [90.8 94.1]	91.6 [89.9 93.3]	0.88 [−1.47 3.22]	0.465
HR (bpm)	74.8 [73.1 76.6]	75.2 [73.4 76.9]	−0.32 [−2.81 2.17]	0.804
SpO_2_ (%)	98.4 [98.3 98.6]	98.4 [98.2 98.6]	0.014 [−0.21 0.23]	0.904

The data were derived from repeated measurements of perioperative hemodynamic parameters in the two patient groups. The group values presented in the table are the estimated marginal means and their 95% confidence intervals (in brackets) calculated using a linear mixed-effects model. The between-group differences (C-E group minus P-E group) are also presented as point estimates with their 95% confidence intervals. Statistical analysis showed no statistically significant differences between the two groups for any of the assessed hemodynamic parameters, including systolic blood pressure (SBP), diastolic blood pressure (DBP), mean arterial pressure (MAP), heart rate (HR), and peripheral oxygen saturation (SpO_2_) (all *p* > 0.05). *p* values are from superiority tests; non-inferiority margins were not pre-specified.

SBP, systolic blood pressure; DBP, diastolic blood pressure; MAP, mean arterial pressure; HR, heart rate; SpO_2_, peripheral oxygen saturation.

For DBP, the estimated marginal means were 73.7 mmHg (95% CI: 72.3–75.2) in the C-E group and 71.9 mmHg (95% CI: 70.4–73.3) in the P-E group. The main effect of group was not statistically significant (*p* = 0.066), nor was the group × time interaction (*p* = 0.075). However, post-hoc analyses revealed that the between-group difference in change from baseline (T0) was significant at T2 (estimate = 3.18, *p* < 0.05) and T3 (estimate = 2.77, *p* < 0.05) (Figure 2B; Table 3).

3. Mean arterial pressure (MAP).

The estimated marginal means of MAP were 92.5 mmHg (95% CI: 90.8–94.1) in the C-E group and 91.6 mmHg (95% CI: 89.9–93.3) in the P-E group. Although the group × time interaction was not statistically significant overall (*p* = 0.117), the between-group difference in change from baseline reached significance at T2 (estimate = 3.29, *p* < 0.05) (Figure 2C; Table 3).

4. Heart rate (HR).

The estimated marginal means of heart rate were 74.8 bpm (95% CI: 73.1–76.6) in the C-E group and 75.2 bpm (95% CI: 73.4–76.9) in the P-E group. Neither the main effect of group (*p* = 0.804) nor the group × time interaction (*p* = 0.282) was significant (Figure 2D; Table 3).

5. Oxygen saturation (SpO₂).

Both groups exhibited similar levels and trends in SpO₂, with an overall mean of 98.4%. No significant main effect of group (*p* = 0.904) or group × time interaction (*p* = 0.366) was detected (Figure 2E; Table 3).

### Recovery quality

The results showed no significant differences between the C-E group and the P-E group in the CFQ Bimodal Score (*p* = 0.701) or the CFQ Likert Score (*p* = 0.746). However, the proportion of patients with a CFQ Bimodal Score ≥4 was significantly lower in the C-E group than in the P-E group (*p* = 0.002). No significant difference was observed between the two groups in the proportion of patients with a CFQ Likert Score ≥18 (*p* = 0.495) (Table 4).

**Table 4:** Recovery quality.

CFQ score	C-E group	P-E group	*p* value
CFQ Bimodal Score (OAA/S ≥ 4)	1.00 [0.00;2.00]	1.00 [0.00;2.00]	0.701
CFQ Likert Score (OAA/S ≥ 4)	12.0 [11.0;13.0]	12.0 [11.0;13.0]	0.746
CFQ Bimodal Score ≥4(yes/no)	11/102	29/81	0.002
CFQ Likert Score ≥18(yes/no)	3/110	5/105	0.495

All patients were assessed using the Chandler Fatigue Questionnaire (CFQ) after regaining consciousness to an OAA/S ≥ 4. CFQ Bimodal Scores and CFQ Likert Scores are presented as median [interquartile range], and intergroup comparisons were performed using the Mann–Whitney U test. The proportions of patients reaching preset thresholds on the CFQ scores are presented as number of events/number of non-events, and intergroup comparisons were performed using the Chi-square test. The results showed no significant differences between the C-E group and the P-E group in the CFQ Bimodal Score (*p* = 0.701) or the CFQ Likert Score (*p* = 0.746). However, the proportion of patients with a CFQ Bimodal Score ≥4 was significantly lower in the C-E group than in the P-E group (*p* = 0.002). No significant difference was observed between the two groups in the proportion of patients with a CFQ Likert Score ≥18 (*p* = 0.495). *p* values are from superiority tests; non-inferiority margins were not pre-specified.

CFQ, Chandler Fatigue Questionnaire; OAA/S, Observer’s Assessment of Alertness/Sedation Scale.

### Adverse events

Hemodynamically significant fluctuations in mean blood pressure (exceeding 20% from baseline) occurred less frequently in the C-E group (C-E: 46/113 vs. P-E: 65/110; *p* = 0.009). One patient in the P-E group experienced a pronounced oxygen desaturation (SpO₂ nadir of 85%) that required intervention with respiratory stimulants and manual ventilation. Meanwhile, fewer patients in the C-E group reported postoperative dizziness at an OAA/S score ≥4 (C-E: 28/113 vs. P-E: 51/110; *p* = 0.001). The occurrence of transient sinus bradycardia was low and not significantly different between groups (C-E: 7/113 vs. P-E: 2/110; *p* > 0.05). The use of vasoactive drugs and respiratory stimulants (excluding the one severe case) was not statistically different. The incidence of post-procedural sore throat (OAA/S ≥ 4) and nausea(OAA/S ≥ 4) were low and comparable between groups (C-E: 7/88 vs. P-E: 3/96, *p* = 0.206; C-E: 1/113 vs. P-E: 0/110, *p* = 1.000) (Table 5).

**Table 5:** Incidence of peri-procedural adverse events.

Adverse events	C-E group	P-E group	*p* value
MAP fluctuations exceeding 20% (yes/no)	46/67	65/45	0.009
hypoxemia (<90%) (yes/no)	0/113	2/108	0.242
transient sinus bradycardia	7/113	2/110	0.111
Use of vasoactive drugs during endoscopy (yes/no)	0/113	1/109	0.493
Use of respiratory stimulants during endoscopy (yes/no)	4/109	9/101	0.233
Mandibular support to open the airway (yes/no)	4/109	9/101	0.233
Severe body movement during endoscopy	7/106	12/98	0.307
Incidence of airway reflexes	1/109	2/107	0.622
Dizziness (OAA/S ≥ 4) (yes/no)	28/85	51/59	0.001
Nausea (OAA/S ≥ 4) (yes/no)	1/113	0/110	1.000
Post-procedural sore throat (yes/no)	7/88	3/96	0.206

Two hundred twenty-three patients received the sedation regimens according to randomization and completed the examination. All patients received sedation regimens according to randomization and completed the endoscopic examination. Intergroup comparisons revealed significant differences in the incidence of mean arterial pressure fluctuations exceeding 20% (*p* = 0.009) and dizziness (*p* = 0.001). However, no statistically significant differences were observed in the incidence of other adverse events, including hypoxemia (<90%), transient sinus bradycardia, intra-procedural use of vasoactive drugs/respiratory stimulants, mandibular support, severe body movement, airway reflexes, nausea (with OAA/S ≥ 4), and post-procedural sore throat.

Airway reflexes, Transient coughing, no procedure interruption; Severe intraoperative body movement indicates the need for additional anesthetic administration.

MAP, mean arterial pressure; OAA/S, Observer’s Assessment of Alertness/Sedation Scale.

Data are presented as number of events/number of non-events. All *p* values were calculated using the Chi-square test. *p* values are from superiority tests; non-inferiority margins were not pre-specified. *p < 0.05 was considered statistically significant.

## Discussion

This study is an exploratory, hypothesis-generating pharmacologic comparison aiming to preliminarily explore the efficacy and safety of the ciprofol-etomidate (C-E) combination for sedation during gastrointestinal endoscopy. The goal of procedural sedation is to suppress the stress response induced by endoscopic stimulation, ensure the safety of the respiratory and circulatory systems, minimize complications, and promote rapid patient recovery and safe discharge (11, 12).

Propofol remains the gold standard for sedation due to its rapid onset and recovery; however, its risks of respiratory depression and hemodynamic fluctuations cannot be ignored (13–15). Etomidate is renowned for its excellent hemodynamic stability but can cause side effects such as myoclonus (16–18). In clinical practice, the two are often combined to leverage their complementary benefits, and this combination has been proven to provide effective and safe sedation (17, 19).

Ciprofol, a structural analog of propofol, is associated with less respiratory depression, minimal injection pain, and improved hemodynamic stability (3, 4). However, direct comparative data on its combination with etomidate remain limited, which is the core motivation for this exploratory study.

The exploratory results of this study indicate that the C-E regimen was comparable to the propofol-etomidate (P-E) regimen in terms of sedation success rate, systolic blood pressure, heart rate, and SpO₂. However, the C-E group demonstrated a more favorable hemodynamic profile, with a significantly smaller decrease in diastolic and mean arterial pressure over time compared to the P-E group. This advantage stems from ciprofol’s milder suppression of central sympathetic activity, which better preserves peripheral vascular tone, and its myocardial contractility suppression, which is only one-third that of propofol (3, 11, 20–22). For patients with cardiovascular comorbidities or the elderly, reducing blood pressure fluctuations is inherently clinically significant, as it may lower the risk of organ hypoperfusion.

Regarding respiratory safety, although the overall incidence of hypoxemia showed no statistical difference, it is noteworthy that two patients in the P-E group experienced SpO₂ below 90% (one dropping to 85% requiring manual ventilation), while no such severe event occurred in the C-E group. This observation aligns with the findings of Karamnov et al. (23, 24), suggesting that the C-E combination may inherently confer a lower risk of respiratory depression. Mechanistically, ciprofol has a weaker relaxant effect on pharyngeal muscles and causes less direct suppression of the respiratory center compared to propofol (3, 8). Combined with etomidate’s minimal respiratory effects, this reduces the risk of tongue retraction, airway collapse, and apnea.

In terms of recovery quality, the incidence of dizziness at recoverable consciousness levels (OAA/S score ≥4) was significantly lower in the C-E group, and the proportion of patients reaching a clinically meaningful state of fatigue (CFQ Bimodal score ≥4) was also smaller. This may be attributed to ciprofol’s more stable hemodynamic profile, avoiding the post-emergence hypotension associated with propofol-induced peripheral vasodilation and the consequent reduction in cerebral blood flow (25). Furthermore, ciprofol’s more gradual decline in plasma concentration and its anti-inflammatory properties may also contribute to improved recovery quality (21, 26, 27). No serious adverse events such as muscle tremors were reported with either combination regimen, indicating good overall safety.

This study has several limitations in addition to retrospective registration. First, it was a single-center study, and the sample size may have limited the detection of certain rare adverse events. Second, the depth of sedation was monitored based on clinical signs and vital parameters rather than objective indicators like electroencephalogram (EEG) monitoring. Third, intravenous bolus injection was used instead of target-controlled infusion, which may affect drug concentration stability. Finally, the study population was limited to individuals of Asian descent. Future research should validate these exploratory findings through multicenter, prospective, pre-registered confirmatory trials, employ objective sedation depth monitoring, conduct pharmacokinetic analyses, and include more diverse populations. Additionally, subsequent studies should implement proper alpha allocation, outcome-specific power calculation, and hierarchical testing to meet the standards of confirmatory trials.

## Conclusion

This single-center prospective randomized controlled exploratory study preliminarily compared the efficacy and adverse effects of ciprofol-etomidate (C-E) versus the commonly used propofol-etomidate (P-E) regimen for sedation during gastrointestinal endoscopy. As a retrospective registered study, it is strictly positioned as an exploratory, hypothesis-generating pharmacologic comparison, and all findings require validation through subsequent prospective, pre-registered confirmatory trials. The exploratory results suggest that the C-E protocol provides a safe and effective sedation option for low-risk patients (ASA I ~ II) undergoing endoscopic procedures, with potential advantages including improved respiratory and hemodynamic stability, consistent sedation depth, reduced postoperative fatigue, and enhanced patient satisfaction. These findings provide valuable hypotheses and data support for future confirmatory research, but they should not be interpreted as confirmatory or causal evidence for clinical practice.

## Data Availability

The raw data supporting the conclusions of this article will be made available by the authors, without undue reservation.
